# Kombucha electronics: electronic circuits on kombucha mats

**DOI:** 10.1038/s41598-023-36244-8

**Published:** 2023-06-09

**Authors:** Andrew Adamatzky, Giuseppe Tarabella, Neil Phillips, Alessandro Chiolerio, Pasquale D’Angelo, Anna Nikolaidou, Georgios Ch. Sirakoulis

**Affiliations:** 1 Unconventional Computing Laboratory, University of the West of England, Bristol, UK; 2 Institute of Materials for Electronic and Magnetism, National Research Council (IMEM-CNR), Parma, Italy; 3 Istituto Italiano di Tecnologia, Center for Converging Technologies, Soft Bioinspired Robotics, Via Morego 30, 16165 Genova, Italy; 4 Department of Electrical and Computer Engineering, Democritus University of Thrace, Xanthi, Greece

**Keywords:** Biomaterials, Materials for devices, Soft materials, Structural materials

## Abstract

A kombucha is a tea and sugar fermented by over sixty kinds of yeasts and bacteria. This symbiotic community produces kombucha mats, which are cellulose-based hydrogels. The kombucha mats can be used as an alternative to animal leather in industry and fashion once they have been dried and cured. Prior to this study, we demonstrated that living kombucha mats display dynamic electrical activity and distinct stimulating responses. For use in organic textiles, cured mats of kombucha are inert. To make kombucha wearables functional, it is necessary to incorporate electrical circuits. We demonstrate that creating electrical conductors on kombucha mats is possible. After repeated bending and stretching, the circuits maintain their functionality. In addition, the abilities and electronic properties of the proposed kombucha, such as being lighter, less expensive, and more flexible than conventional electronic systems, pave the way for their use in a diverse range of applications.

## Introduction

Kombucha is fermented by a symbiotic community of bacteria and yeasts^1–5^. The symbiotic culture of bacteria and yeasts produces a cellulose-based hydro-gel, also known as bacterial cellulose, biofilm, commensal biomass, tea-fungus, scoby and zooglea. A tea fermented by the symbiotic community allegedly exhibits a range of health beneficial properties^2,6,7^, however these will not be discussed in the present work.

Kombucha mats are unique symbiotic systems where over sixty species of yeasts and bacteria cooperate^1^. A kombucha is an example of a proto-multicellularity—an organism combined of multiple species each one pursuing a common goal of prolonging a life time of the collective organism. Electrical properties of kombucha mats, firstly uncovered in^8^, can further advance ideas on electricity based integration, and possibly, protocognition of symbiotic organisms^9–12^. Similar bacterial cellulose mats, for example, produced by *Acetobacter aceti* colonies, have been shown to feature interesting electrical properties and pressure sensing capabilities^13^.

Kombucha mats, when properly cured, show properties similar to textiles^14–19^, and might make a competitive alternative to fungal leather and wearables^20,21^.

Wearables made of kombucha, while not a commonly known concept, could potentially offer several benefits. Kombucha forms a cellulose-based mat on the surface. This cellulose material has unique properties that make it a promising candidate for wearable technology. Here are some reasons why wearables made of kombucha could be important:

- Sustainability: Kombucha wearables could be more sustainable compared to traditional wearable materials. The cellulose-based material is biodegradable, renewable, and can be grown using simple ingredients like tea and sugar. It has the potential to reduce the environmental impact associated with the production and disposal of traditional wearables made from synthetic materials.
- Biocompatibility: The cellulose material derived from kombucha is generally biocompatible, meaning it is less likely to cause adverse reactions when in contact with human skin. This makes it a potentially suitable material for individuals with sensitive skin or allergies.
- Customizability: The kombucha material can be molded into various shapes and sizes during its growth process, allowing for customized wearables that can conform to individual body shapes and needs. This flexibility could lead to improved comfort and performance.
- Breathability and Moisture Management: Kombucha-based wearables have the potential to be highly breathable, allowing air circulation and reducing moisture buildup on the skin. This property could be beneficial for athletic wear or other applications where moisture management is important. Moreover, water uptake of kombucha will have effects on the increasing of kombucha bulk conductivity; the adhesion to skin is also increased after sweat absorption, making possible self-adhesion patches made of vegetable materials.
- Sensor Integration: Kombucha wearables could potentially incorporate sensors and electronics within the material itself, providing a seamless and unobtrusive integration of technology with the human body. This could open up new possibilities for monitoring health metrics, tracking movement, or providing haptic feedback.

It’s worth noting that while the concept of wearables made of kombucha holds promise, it is still an emerging area of research and development. Challenges related to durability, scalability, and mass production would need to be addressed before such wearables become commonplace. However, the potential for sustainability and unique material properties make kombucha wearables an intriguing prospect for the future.

In a light of ongoing research on sensing and computing mechanisms embedded in living wearables^22–25^ we aim to evaluate kombucha zoogleal mats as potentially embeddable cyber-physical wearable devices with non-linear and non-trivial electrical properties. To achieve the aim we test if basic components of the electrical circuits could made on dry kombucha mats.

Modern electrical circuits require reliable electrical connections between electronic components (including sensors) and external signals for their construction and continued operation^26–28^. Printed circuit boards (PCBs) are typically constructed from silkscreen, solder mask, copper, and substrate^29,30^. Material selection is crucial to the successful operation of printed circuit boards, especially thermal behaviour. The majority of PCB substrates fall into one of two categories: hard/rigid or soft/flexible. Ceramic-based materials typically provide excellent thermal conductivity, good dielectric properties, a high operating temperature, and a low expansion coefficient. The most popular rigid material is FR-4, a glass-reinforced epoxy laminate that is both inexpensive and versatile^31,32^. Above a few GHz, the substantial dielectric loss (dissipation factor) of FR-4 renders it unsuitable for high-speed digital or high-frequency analogue circuits^33,34^.

PCBs for wearables should be be mechanically flexible, waterproof, and shockproof and by default light^35–39^. Traditionally they are plastic based although they typically lack sustainability and cost-effectiveness. Polymeric soft materials offer superior resistance to stretching, bending and washing cycles^40^. Moreover, wearables are intended to interact closely with their wearer, therefore bio-compatibility is advantageous, or at least resistance to the active chemical environment offered by the human skin. Therefore, the combination of bio-based PCBs and biodegradable components (including ICs) is especially advantageous for wearables.

## Results

Kombucha mats are proven to be robust to tearing, and are not destroyed even by immersion in water for several days. The mat survived oven temperature up to 200C but burn when exposed to open flame. We have demonstrated that it possible to (1) precisely cut kombucha mats with laser, (2) aerosol jet print PODOT:PSS circuits on kombucha mats, (3) 3D printing TPU and metal-polymer composite on kombucha mats, (4) draw conductive tracks and arrange functional elements with conductive paints.

**Figure 1:**
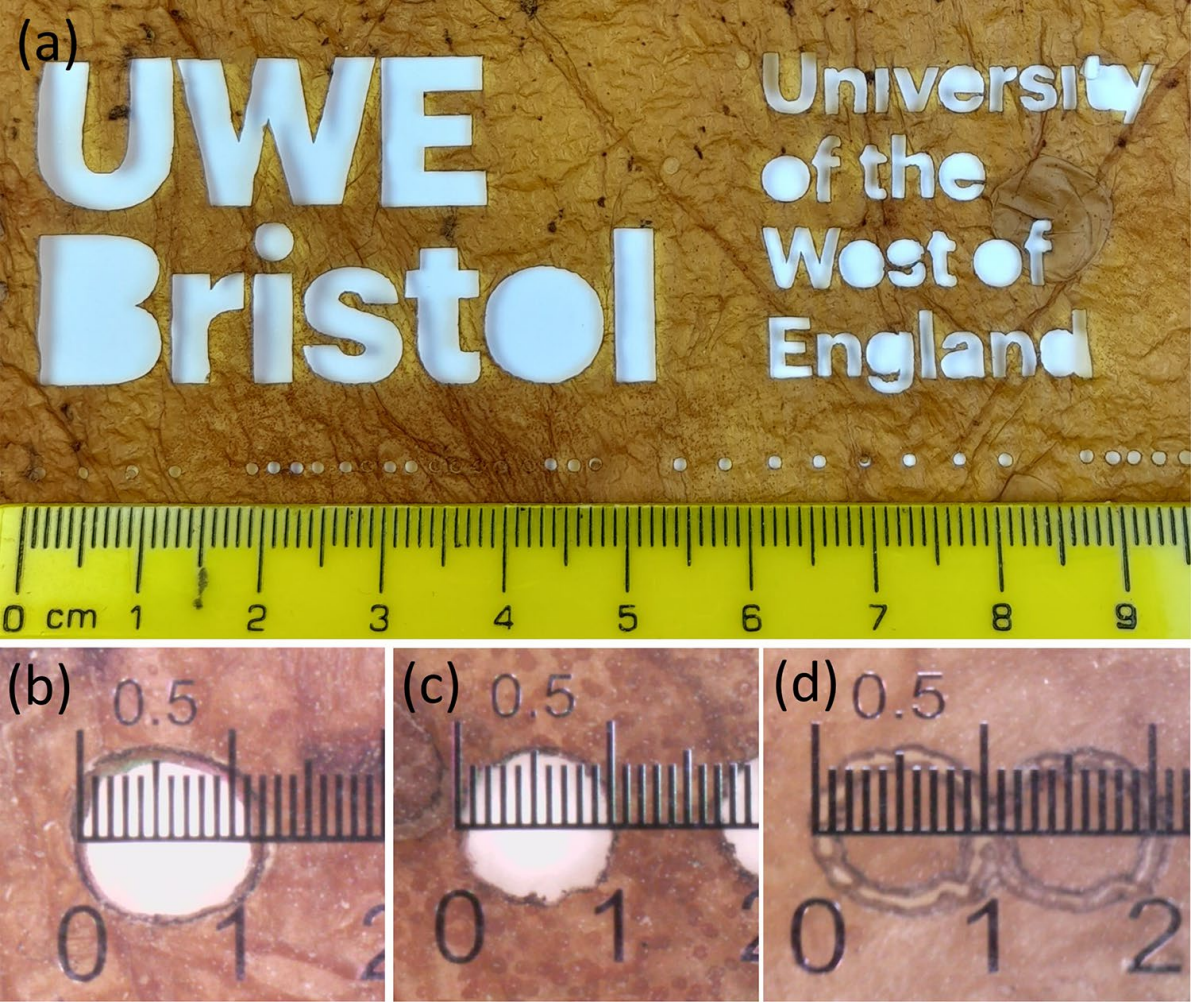
Kombucha mats cut with laser cutter (**a**) letters and holes of different sizes, (**b**) nominal 1 mm hole cut to $\sim$1.1 mm diameter with $\sim$25 W laser power, (**c**) nominal 1 mm hole cut to $\sim$1.0 mm diameter with $\sim$18 W laser power, (**d**) nominal 1 mm holes only partly cut out to $\sim$1.0 mm diameter with $\sim$10 W laser power.

Laser cutting proved to be a problem free procedure. Exemplars of kombucha mats cut with laser cutter are shown in Fig. 1. The laser settings (e.g. speed of motion, beam power and number of laser pulses per inch) were found to be critical to accurate cutting. The optimal setting for 0.45 ± 0.1 mm thickness was found to be 80 inches per second, $\sim$18 W, and 500 pulses per inch, as shown in Fig. 1c. If the beam power is raised above optimal level the cut becomes wider than desirable, as shown in Fig. 1b. Conversely, if the beam power is lower than optimal level the mat is only partly cut through, as shown in Fig. 1d. With optimised settings, kombucha mats were found to cut well with minimal smoke. Some cut sections needed to be agitated free for removal.

Organic electrical conductors have been printed by Aerosol Jet Printing (AJP) with the aim of creating circuits over kombucha mats, exploited as potential substrates in wearable electronics. Circuits over kombucha can act in perspective as sensors or biosensors, coupled also with printed antennas for wireless data communication and storing in clouds. Herein, we are going to explore basic properties of printed traces over the surface of kombucha.

Aerosol Jet Printing is particularly suitable for printing over irregular surfaces, flexible and/or stretchable substrates made of natural materials (bio-polymers) because of it operates in non-contact mode at a fixed distance from the substrate. Basic principles and mechanisms of AJP techniques have been discussed in literature^41–44^. This technology belongs to the additive manufacturing sector and offers advantages with respect to other well known technologies and broadly distributed, such as ink jet printing (normally referred to liquid inks jetted using thermal or piezoelectric nozzles^45^).

A highly conductive formulation of PEDOT:PSS was used as ink: 2mL of ink was uploaded in the ultrasonic atomiser of AJP 200, by setting the gas flows at 30 and 25 sccm for the atomiser and sheath gas, respectively. A 200 um size nozzle was mounted on the printed head. The printing run was operated on cool-conditions to avoid exposure of kombucha to heat treatments. Elementary circuit elements were printed firstly, 3 circular electrodes (2 mm diameter) at a fixed distance, acting as the working, counter and reference electrodes, for the evaluation of impedance of the electrode-kombucha interface, by Impedance Electrochemical Spectroscopy (EIS) analysis.

**Figure 2:**
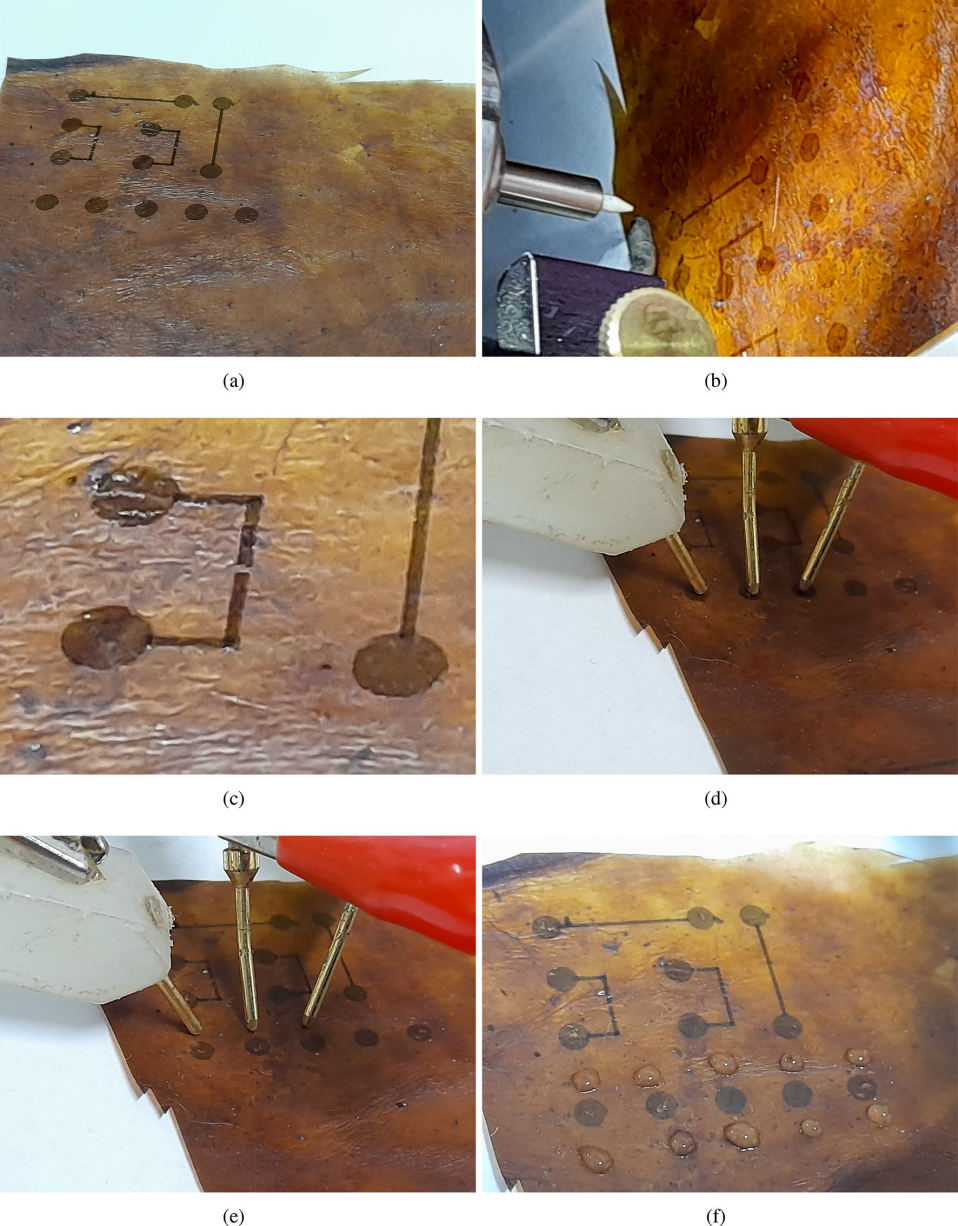
Exemplars of deposition of PEDOT:PSS circuits and measurements of electrical properties (**a**) PEDOT:PSS round pads at a fixed distance from each other with interconnecting tracks (**b**) Aerosol Jet Printing nozzle (**c**) defined gap between tracks (**d**) spring loaded electrodes on PEDOT:PSS pads (**e**) spring loaded electrodes on surface of kombucha (**f**) hydration of PEDOT:PSS.

**Figure 3:**
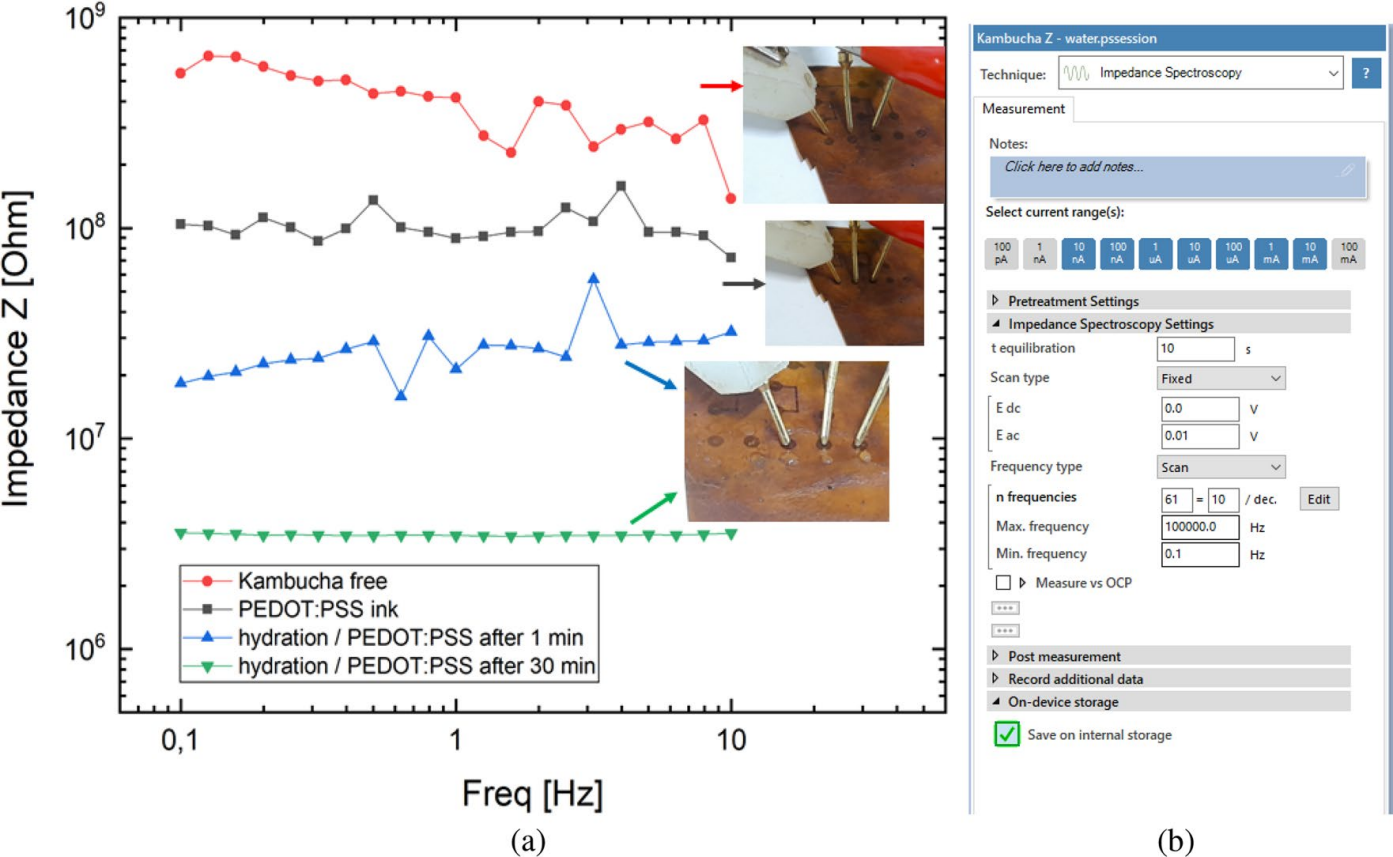
Electrical properties of kombucha mat with and without PEDOT:PSS circuits (**a**) impedance against frequency (**b**) spectroscopy settings.

Exemplars of deposition of PEDOT:PSS circuits and measurements of electrical properties are shown in Fig. 2. The figure shows the acquired data of EIS over (1) three free points over the kombucha surface; (2) three PEDOT:PSS electrodes used as the working (RE), counter (CE) and reference (RE) electrodes, placed at fixed distances and the same as the free points of (1); (3) the same measurements of (2) after hydration, where hydration was performed by placing 20 $\upmu L$ of water drops in the surrounding area of the electrodes over the kombucha surface. Being kombucha a cellulose-based material, it is very sensitive to water absorption, and up-taking water in the kombucha backbone make the kombucha foil more conductive. Impedance measurements increase almost instantly after water dropping, and stabilise quickly; the measurements after 30 min after water dropping show a more stable signal. Electrical properties of kombucha mat with and without PEDOT:PSS circuits are shown in Fig. 3.

**Figure 4:**
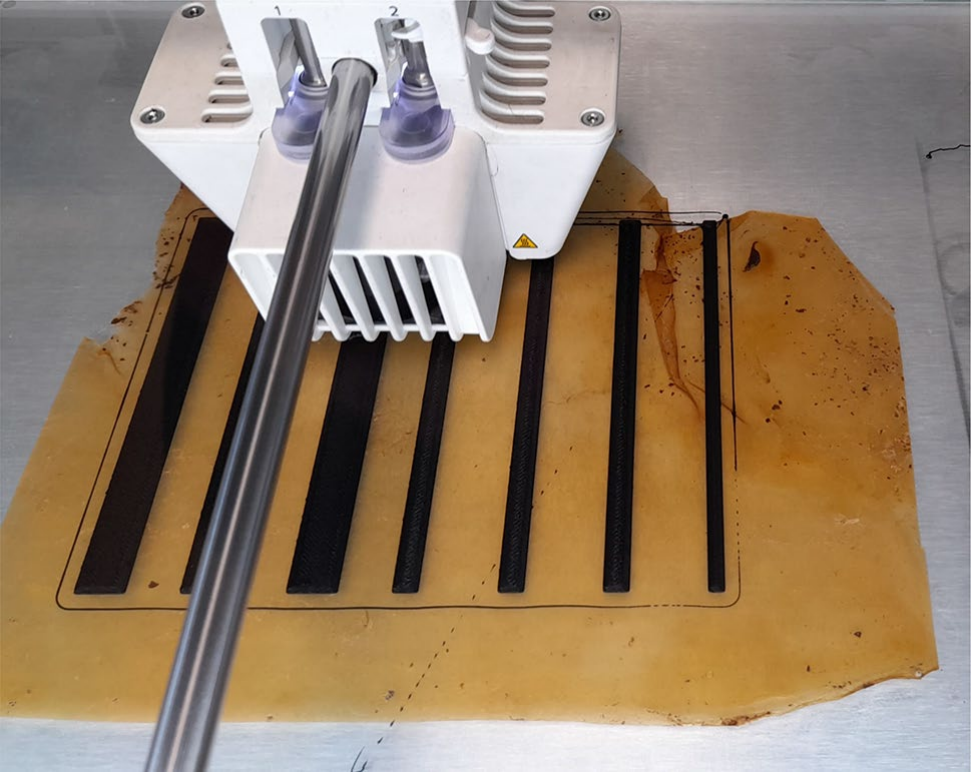
3D printing of flexible TPU (with 15% carbon infill) tracks on kombucha mat.

Exemplar of tracks of TPU (with 15% carbon infill) 3D printed on kombucha mat are shown in Fig. 4. Track resistance of TPU (with 15% carbon infill) and Electrifi (metal-polymer composite—biodegradable polyester and copper) was found to vary with width and thickness,as summarised in Table 1. Tracks of 100 mm length were measured with LCR meter (891, BK Precision, UK). Flexibility of the tracks was found to vary with thickness. The performance of each manufacturing method is linked to capabilities of the constituent material(s) used. For example, TPU is a flexible elastomer that offers high mechanical strength, good chemical resistance, and excellent abrasion resistance. Furthermore, TPU has a strong adherence to a variety of substrates and may be easily processed utilising a variety of techniques such as injection moulding and extrusion. By mixing with carbon it can become electrically conductive. However, the electrical conductivity of TPU remains lower than desirable despite carbon loading. Conversely, Electrifi has good electrical conductivity but poorer mechanical properties. Silver-loaded inks provide acceptable conductivity for some applications, excellent adhesion to a variety of substrates, and a modest cost. However, there are some drawbacks to using silver-loaded inks, such as the tendency to oxidise when exposed to air, resulting in a decrease in conductivity over time. Despite these drawbacks, silver-loaded ink remains a popular choice for printed electronics due to its ease of processing and printing. Furthermore, progress has been made in developing silver nanoparticle-based ink with improved electrical conductivity that can be used in a variety of applications such as biosensors and stretchable electronics.

Both TPU and Electrifi tracks remained attached to kombucha after a couple of days of immersion in water. Their attachment might be ’mechanical’ rather than chemical as the liquid (melted) polymers are effectively ’injected’ into/onto the surface of kombucha effectively filling any surface irregularities which then act as ’grips’ holding track in position. The formulation of flexible and stretchable inks is an active area of research where many efforts are in progress. New experimental metal inks formulations with stretchable and flexible properties have been proposed recently, applied in inkjet and aerosol jet printing. Tracks printed with these new inks would have the potential to adapt and follow the stretching and bending of the underneath substrates. We are in progress to test some of these new formulation and to make home-made inks as well.

**Table 1:** Track resistance of TPU (with 15% carbon infill) and Electrifi.

Width	Thick-ness	Cross-section	TPU with 15% carbon	Electrifi
(mm)	(mm)	(mm$^2$)	($\Omega \hbox {cm}^{-1}$)	($\Omega \hbox {cm}^{-1}$)
10	5	50	650	
10	3	30	920	
10	1	10	2450	
5	3	15	1400	
5	2	10	2120	0.28
5	1	5	3440	0.43
3	1	3	4490	0.89

**Figure 5:**
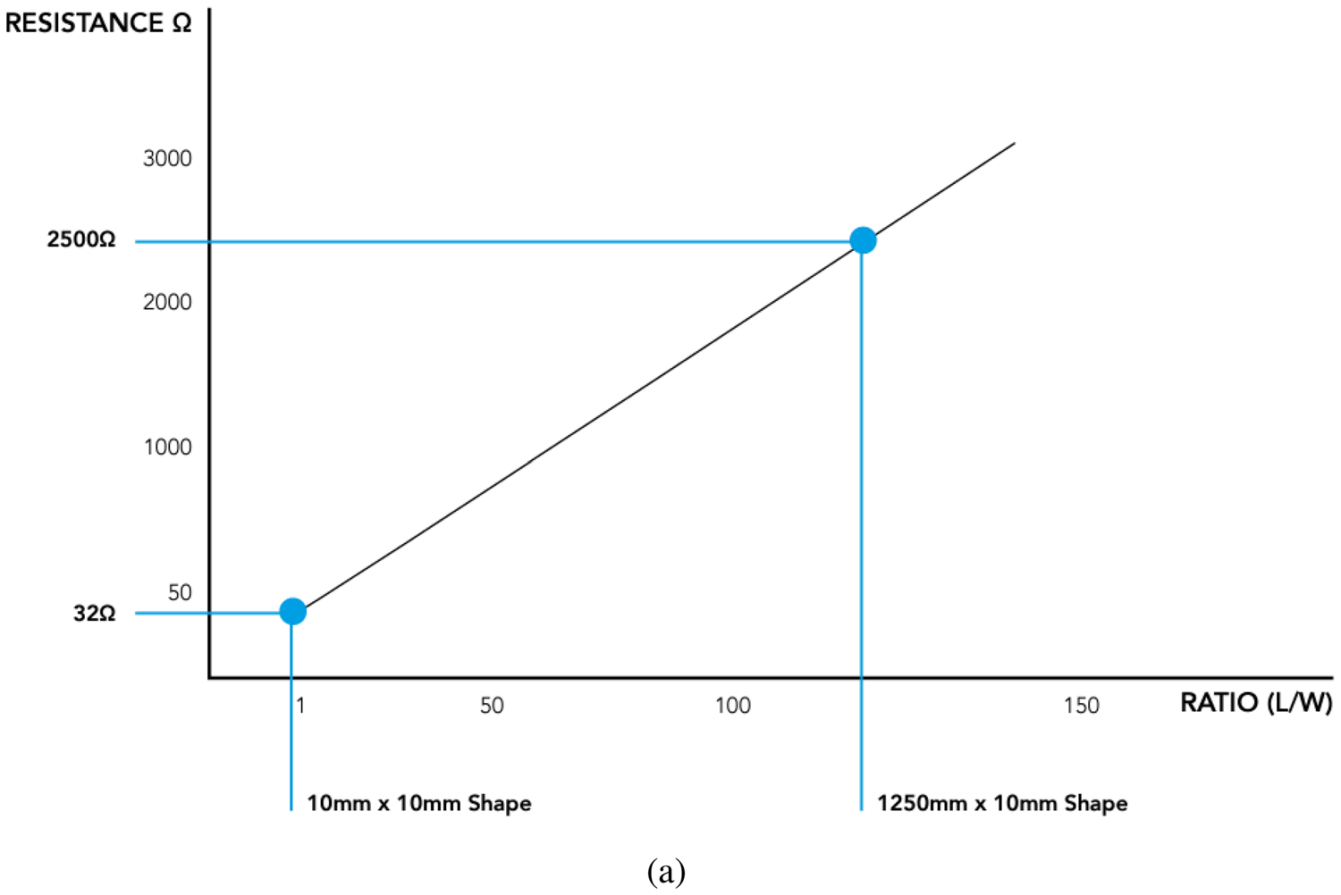
Electrically conductive paint, data-set from Bare Conductive (UK).

With regards to electrically conductive paint, experiments demonstrated that ‘Bare Conductive’^46^ adheres well to the kombucha mats and sustains some degree of flexibility. Typical electrical conductivity for tracks is shown in Fig. 5. Track resistance of the conductive paint tracks on kombucha mats varied between 20 $\Omega \hbox {cm}^{-1}$ to 200 $\Omega \hbox {cm}^{-1}$. These values roughly align with the ‘Bare Conductive’ data-sheet^46^ with ‘thick’ tracks. Track resistance of XD-120 conductive silver ink on kombucha mat was also found to vary. Typical range 1.5 $\Omega \hbox {cm}^{-1}$ to 10 $\Omega \hbox {cm}^{-1}$

## Discussion

Four technologies for manufacturing kombucha based PCBs were explored aerosol jet printing of PODOT:PSS, 3D printing of TPU and metal-polymer composite, adding ink with conductive filler and laser cutting. Each offered advantages and disadvantages compared to other technologies.

**Figure 6:**
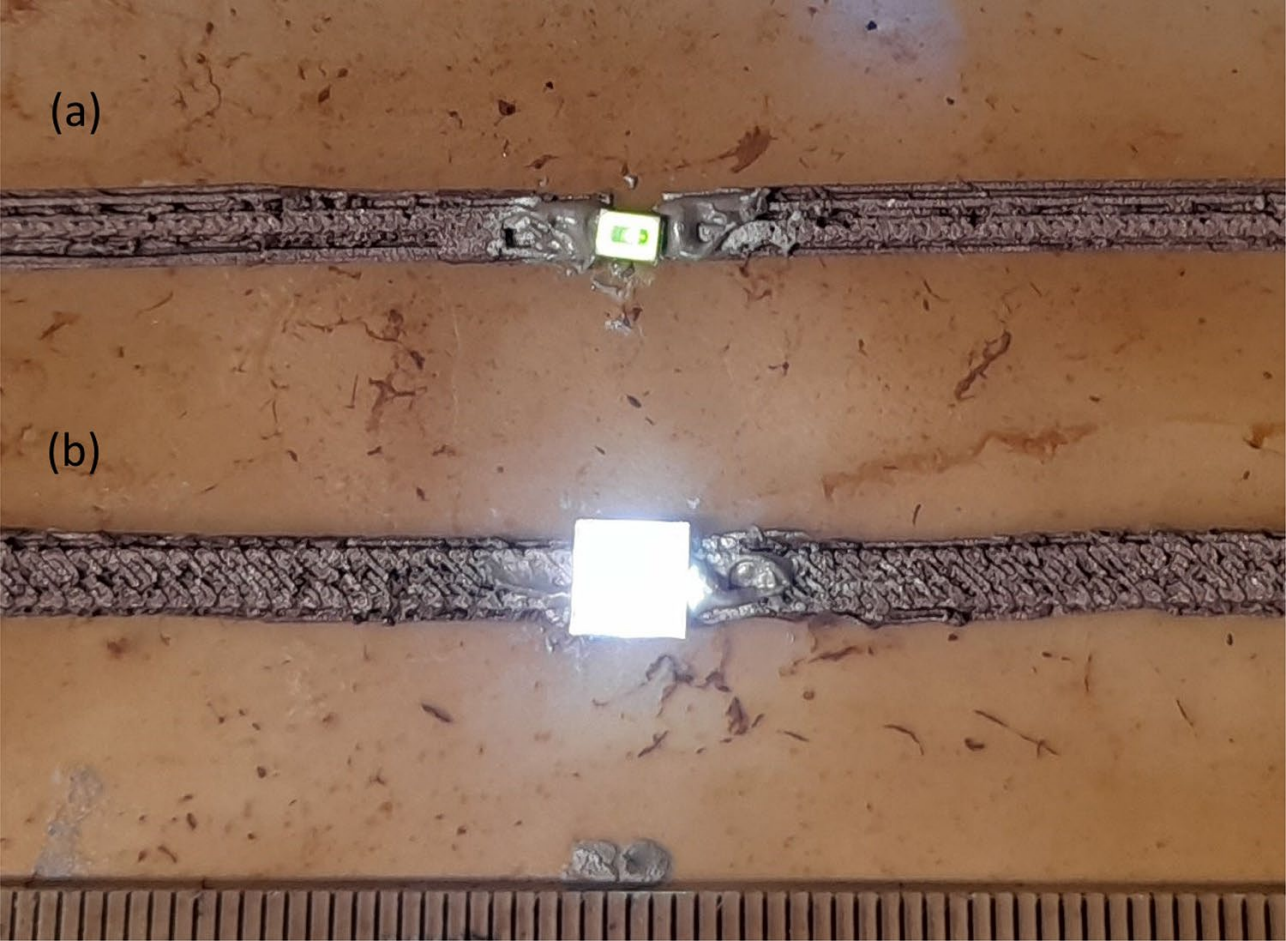
Exemplar of metal-polymer composite (Electrifi) tracks on kombucha mat (**a**) $\sim$3 mm wide track with SMD LED (3020 package) green colour (**b**) $\sim$5 mm wide track with SMD LED (5050 package) white colour (scale of ruler in mm).

As demonstrated in Fig. 6, it is feasible to construct electrical circuits on kombucha mats. Two track widths ($\sim$3 and $\sim$5 mm) and two packages (3020 and 5050) of surface mount devices (SMD) are displayed. A silver-loaded, conductive, two-part epoxy (Chemtronics CW2400^47^) was manually applied to mechanically attach and electrically connect SMDs to polymer tracks. For volume manufacture, SMDs would be automatically mounted using a pick and place machine and conductive epoxy precisely and automatically dispensed with in-line dispensers.

**Figure 7:**
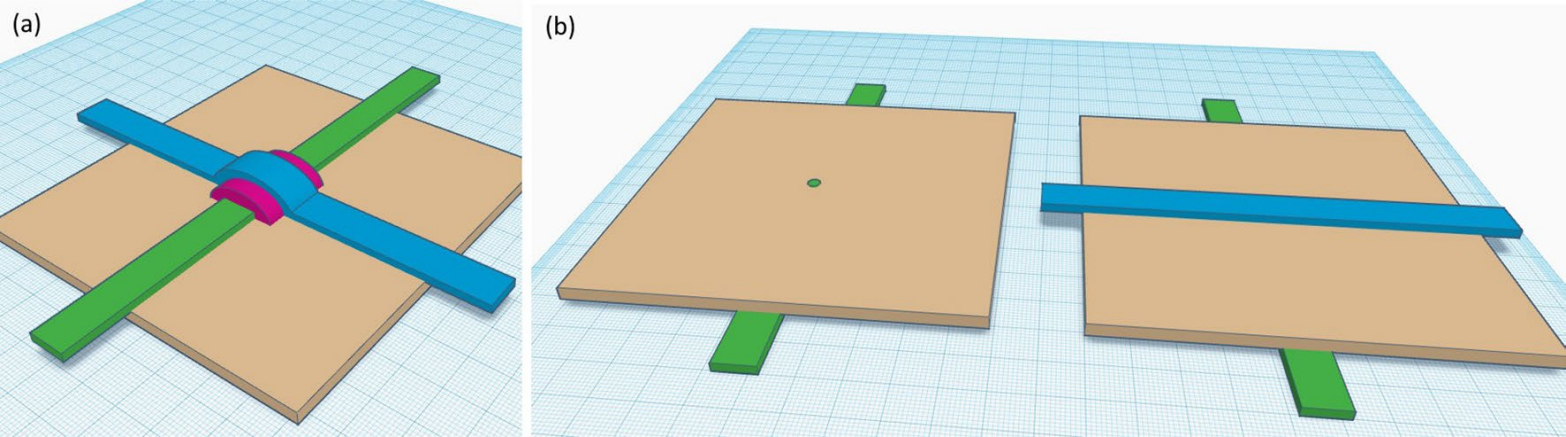
Methods of cross connecting on kombucha mats (**a**) single sided cross-over bridge with insulator between (**b**) through-hole double sided with laser hole cutting.

Two potential methods of forming cross connections on kombucha mats via 3D printing of conductive material—single sided cross-over bridges and through-hole double sided via laser hole cutting—are illustrated Fig. 7.

Kombucha mats show properties that can be exploited to envision potential and future kombucha-based devices. The hydration-dependent electrical conduction of kombucha allows to extend the potential operational frequency range of surface-electrodes over kombucha mats, as well as to exploit the kombucha mat as a resistive switching device in a planar electro-chemical cell. High-quality kombucha mat production necessitates quality control measures to ensure purity and consistency. One of the most important quality control measures for bacterial cellulose production is ensuring that the culture medium used for production is free of contaminants. Contaminants can have a significant impact on the quality of bacterial cellulose, resulting in inconsistent results. Another important quality control measure is the use of standardised protocols for bacterial cellulose harvesting and purification. This includes monitoring the pH, temperature, and bacterial growth during the manufacturing process. Quality of the kombucha mats produced can be controlled by adjusting liquid temperature and concentration of nutrients, following published protocols^48–50^.

Future research will be concerned with printing advanced functional circuits, capable for detecting, and may be recognising, mechanical, optical and chemical stimuli, implementing sensorial fusion and distributed information processing.

## Methods and materials

**Figure 8:**
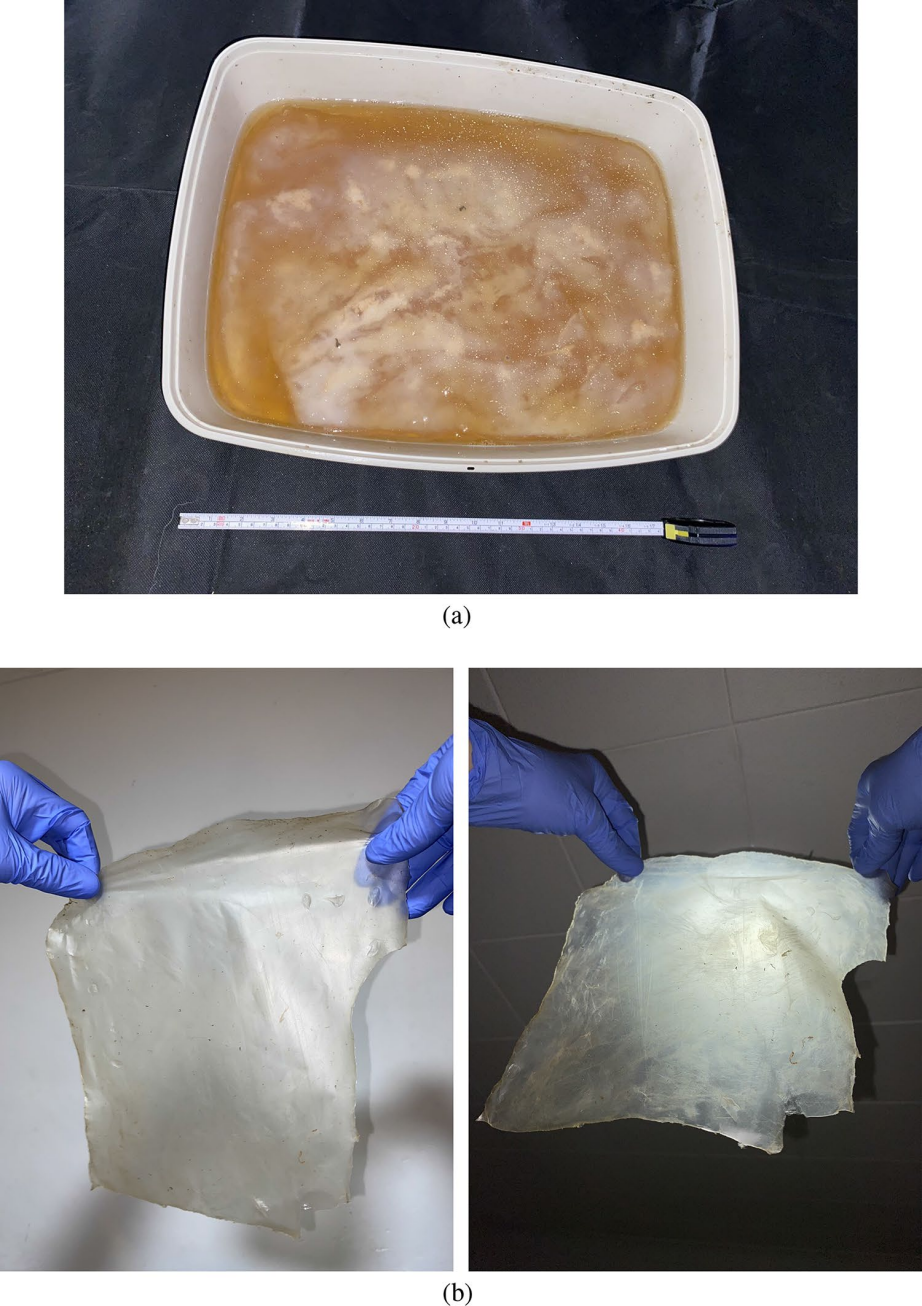
(**a**) Container with kombucha live mat on the surface of the liquid culture. (**b**) Dried mat.

The kombucha zooglea was commercially sourced (Freshly Fermented Ltd, UK) to grow mats of kombucha *in situ*. The infusion was prepared as follows; 2% tea (PG Tips, UK), 5% sugar (Silver Spoon, UK), and 1 L of boiled mains water. Containers with kombucha (Fig. 8) were stored at ambient temperature (20–23$\,^{\circ }$C) in darkness. The solution was refreshed each week. Kombucha mats were removed from the cultivation container and air-dried on plastic or paper at ambient temperature (several techniques were tried).

Four manufacturing technologies to add conductive tracks, attaching electronic components, and cut profiles of kombucha mats were explored.

Aerosol jet printing of PODOT:PSS was implemented as follows. Organic-based electrodes and interconnecting lines were printed by Aerosol Jet Printing (AJP200, Optomec, US^51^) by using an a highly-conductive inkjet formulation of PEDOT:PSS (Clevios P JET N V2, Heraeus, US^52^). Printing parameters were optimised for achieving conductive traces over the surface of kombucha mats used as the substrate. Electrochemical measurements were performed by a potentiostat (PalmSens4, PalmSens BV, NL^53^).

To 3D print TPU with 15% carbon infill and metal-polymer composite—biodegradable polyester and copper—two compositions of filament (2.85 mm diameter) were hot extruded onto kombucha mats via 0.4 mm nozzle on 3D printer (S5, Ultimaker, UK^54^). The composition filaments were ‘Conductive Filaflex Black’ rated 3.9 $\Omega \hbox {cm}^{-1}$^55^ and ‘Electrifi Conductive Filament’ rated 0.006 $\Omega \hbox {cm}^{-1}$^56^.

Conductive pathways were drawn onto kombucha mats with two compositions of conductive ink, including ‘Bare Conductive’ rated 55 $\Omega \hbox {sq}^{-1}$ at 50 ${\upmu }\hbox {m}$ thickness^57^ and ‘XD-120 conductive silver ink’ rated rated 0.00003 $\Omega \hbox {cm}^{-1}$^58^.

When shaping was involved, kombucha mats of $0.45{\pm 0.1}$ mm thickness were cut with 75 W CNC laser cutter (Legend 36EXT, Epiloglasers, US,^59^) while parameters (speed of motion, beam power, pulses per inch) were adjusted to determine the optimal settings.

## Data Availability

The raw data-sets obtained in this study are available from the corresponding author on reasonable request.
