# Author Correction: Kombucha electronics: electronic circuits on kombucha mats

**DOI:** 10.1038/s41598-023-38161-2

**Published:** 2023-07-11

**Authors:** Andrew Adamatzky, Giuseppe Tarabella, Neil Phillips, Alessandro Chiolerio, Pasquale D’Angelo, Anna Nikolaidou, Georgios Ch. Sirakoulis

**Affiliations:** 1 Unconventional Computing Laboratory, University of the West of England, Bristol, UK; 2 Institute of Materials for Electronic and Magnetism, National Research Council (IMEM-CNR), Parma, Italy; 3 Istituto Italiano di Tecnologia, Center for Converging Technologies, Soft Bioinspired Robotics, Via Morego 30, 16165 Genova, Italy; 4 Department of Electrical and Computer Engineering, Democritus University of Thrace, Xanthi, Greece

Correction to: *Scientific Reports* 10.1038/s41598-023-36244-8, published online 09 June 2023

The original version of this Article contained an error in the spelling of the author Pasquale D’Angelo which was incorrectly given as Passquale D’Angelo.

The original Article has been corrected.

